# Stream Physical Characteristics Impact Habitat Quality for Pacific Salmon in Two Temperate Coastal Watersheds

**DOI:** 10.1371/journal.pone.0132652

**Published:** 2015-07-29

**Authors:** Jason B. Fellman, Eran Hood, William Dryer, Sanjay Pyare

**Affiliations:** Environmental Science and Geography Program, University of Alaska Southeast, Juneau, Alaska, United States of America; Northwest Fisheries Science Center, NOAA Fisheries, UNITED STATES

## Abstract

Climate warming is likely to cause both indirect and direct impacts on the biophysical properties of stream ecosystems especially in regions that support societally important fish species such as Pacific salmon. We studied the seasonal variability and interaction between stream temperature and DO in a low-gradient, forested stream and a glacial-fed stream in coastal southeast Alaska to assess how these key physical parameters impact freshwater habitat quality for salmon. We also use multiple regression analysis to evaluate how discharge and air temperature influence the seasonal patterns in stream temperature and DO. Mean daily stream temperature ranged from 1.1 to 16.4°C in non-glacial Peterson Creek but only 1.0 to 8.8°C in glacial-fed Cowee Creek, reflecting the strong moderating influence glacier meltwater had on stream temperature. Peterson Creek had mean daily DO concentrations ranging from 3.8 to 14.1 mg L^−1^ suggesting future climate changes could result in an even greater depletion in DO. Mean daily stream temperature strongly controlled mean daily DO in both Peterson (R^2^=0.82, P<0.01) and Cowee Creek (R^2^=0.93, P<0.01). However, DO in Peterson Creek was mildly related to stream temperature (R^2^=0.15, P<0.01) and strongly influenced by discharge (R^2^=0.46, P<0.01) on days when stream temperature exceeded 10°C. Moreover, Peterson Creek had DO values that were particularly low (<5.0 mg L^−1^) on days when discharge was low but also when spawning salmon were abundant. Our results demonstrate the complexity of stream temperature and DO regimes in coastal temperate watersheds and highlight the need for watershed managers to move towards multi-factor risk assessment of potential habitat quality for salmon rather than single factor assessments alone.

## Introduction

Alaska is a globally important wild-salmon producing region [1], with more than 4,000 natural runs in southeast Alaska alone [2]. Characterizing suitable aquatic habitat for salmon in coastal watersheds is becoming an increasingly important component of watershed management in light of rapid environmental change in the region. The primary drivers of environmental change include: rising air temperatures [3], changes in the rain/snow fraction of precipitation [4] and glacier volume loss [5], all of which have the potential to impact the physical properties of salmon-producing streams. Of particular importance is the potential these environmental changes have for altering thermal regimes in coastal salmon-producing streams in Alaska [6–9].

Temperature is a fundamental control on metabolic activity and the solubility of dissolved gases (e.g., carbon dioxide and oxygen) in streamwater. As a result, shifts in watershed thermal regimes can impact the availability of freshwater habitat for cold-water fish species [10,11]. For salmonids (e.g., salmon and trout), temperatures that exceed their range of tolerance can adversely affect the growth and development, distribution and abundance, migration and behavior at all life stages [12–14]. In addition to temperature, dissolved oxygen (DO) is an important control on inland aquatic habitat for salmonids because DO levels exert a strong influence on salmonid behavior and metabolic activity [13,15]. Low concentrations of DO can be lethal to salmon, however sub-lethal effects are far more common and include impacts to the growth and development of salmon at different life stages, decreased feeding activity, reduced swimming performance and prevention of upstream migration [15–17].

The physical characteristics of streamwater are controlled by numerous factors including air temperature, discharge, streambed processes and topography [10,18,19]. In the Coast Mountains of Alaska, glaciers and seasonal snowcover are particularly important landscape controls on streamwater physical characteristics because meltwater from these cryospheric reservoirs moderates stream temperature during the summer months [8,20–22]. Glaciers can also attenuate the inter-annual variability in discharge by maintaining streamflow during extended periods in the summer runoff season [23,24]. As a result, changes in snow and glacier melt have the potential to substantially influence habitat quality for both adult and juvenile salmon [20] as well as aquatic macroinvertebrates (e.g.,[25]).

To date, many watershed-scale studies of salmon habitat quality have focused on either stream temperature or DO alone [26,27]. However, multi-factor studies that allow for a mechanistic understanding on the controls of both stream temperature and DO are clearly necessary given the importance of these parameters to salmon habitat quality. Moreover, the independent effects of stream temperature and DO on salmon habitat quality are often difficult to untangle given their interaction with each other and other biophysical controls such as discharge. We used a paired watershed approach to assess the seasonal variability and interaction between stream temperature and DO in two salmon bearing watersheds in coastal southeast Alaska that differ in terms of snow and glacier melt inputs to streamflow. Multiple regression analysis was used to evaluate how the climatically sensitive variables of stream discharge and air temperature influence seasonal patterns in stream temperature and DO. We further assess whether spawning salmon abundance influences streamwater DO via biological oxygen demand. Our results provide insight into the potential impacts of climate warming on habitat quality for salmon in coastal temperate watersheds adjacent to the Gulf of Alaska.

## Materials and Methods

### Ethics statement

Field sampling was conducted under a permit obtained from the United States Department of Agriculture, Tongass National Forest.

### Site description

**Fig 1:**
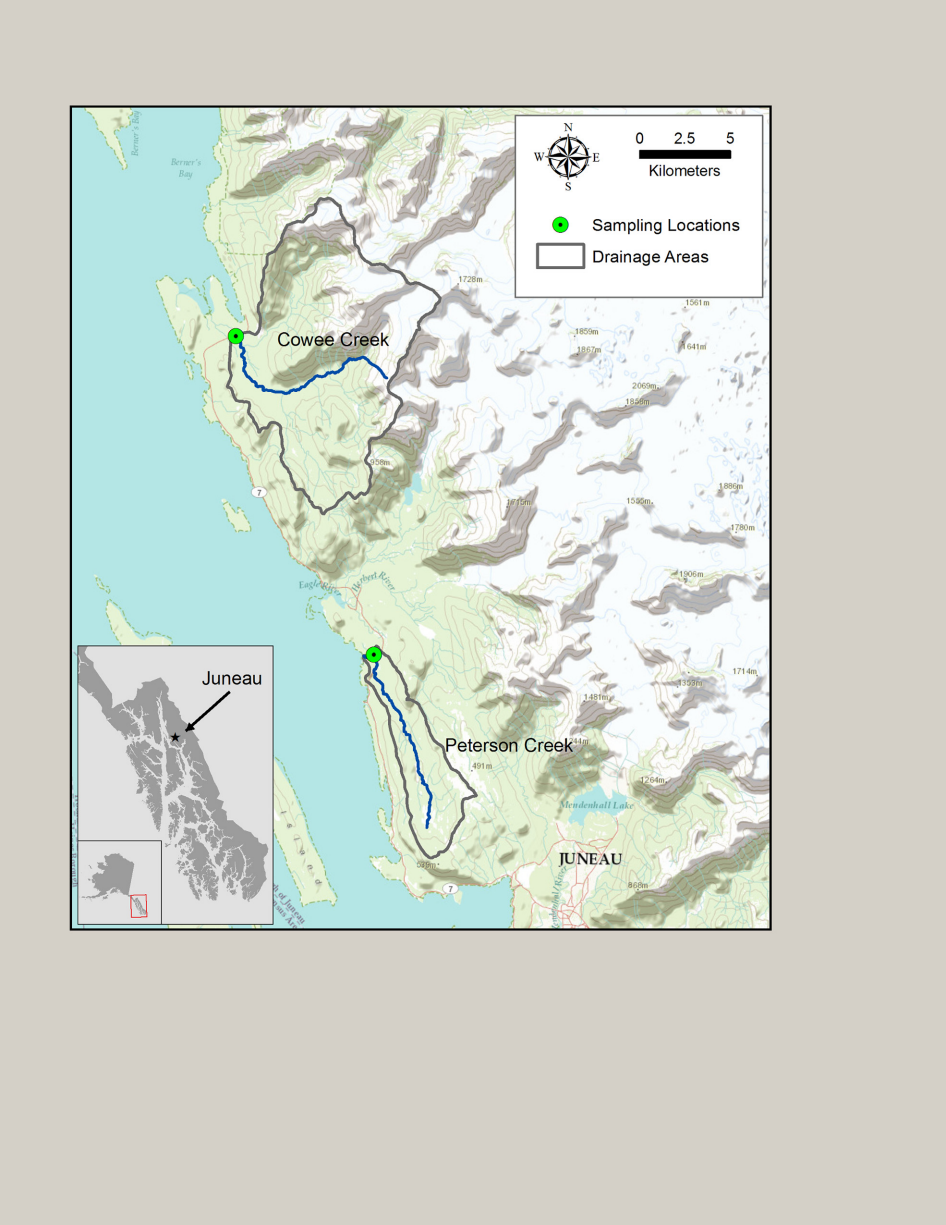
Map of the two study watersheds and sampling sites near Juneau, Alaska.

The two study watersheds are located near Juneau in northern southeast Alaska (Fig 1). Juneau has a maritime climate with mild winters and cool, wet summers and a mean annual precipitation of 1400 mm at sea level. Extensive glaciation has modified the region leaving heavily incised watersheds with steep slopes but also abundant lowlands characterized by peatlands mixed with coniferous forest.

Cowee Creek (watershed area of 110 km^2^) contains a hanging glacier complex with 13% of the watershed covered by glacier ice. Average watershed elevation is 638 m with areas of alpine tundra and exposed bedrock at high elevations and a mixed coniferous forest of *Picea sitchensis* and *Tsuga heterophylla* in the mid to lower watershed. Cowee Creek is low gradient downstream near the monitoring station, located ~4 km from the watershed outlet to Berners Bay. A thick riparian forest occupies most of the mid to lower reaches of the watershed, although the main channel is not heavily shaded with wetted-widths typically 10–15 m. Non-glacial Peterson Creek has a watershed area of 24 km^2^ and a mean watershed elevation of 309 m. Peterson Creek contains large areas of forested and unforested peatland (34% watershed wetland coverage) and has a small lake near its headwaters. The vegetation in the remainder of the watershed is similar to the mid to lower watershed in Cowee Creek consisting mainly of a mixed coniferous forest of *P*. *sitchensis* and *T*. *heterophylla*. The stream channel is partially to heavily shaded, with wetted widths that range from 5–8 m near the monitoring station.

Peterson and Cowee Creeks both have runs of Pacific salmon (*Oncorhynchus kisutch*, *O*. *keta and O*. *gorbuscha*) lasting from late June through mid-October that vary in spawner density. Salmon carcass densities of *O*. *keta* in Peterson Creek during July and August can be as high as 3.5 kg m^-2^ or 0.5 fish m^-2^ [28,29]. In Cowee Creek, salmon carcass densities (mostly *O*. *gorbuscha*) are unknown although they are substantially lower than in Peterson Creek based on frequent observations from previous years and during the study period. Previous research in both streams has shown that salmon deliver large quantities of nutrients and organic matter to streamwater, particularly during periods of high spawner density in Peterson Creek and to a lesser extent Cowee Creek [28–30].

### Field methods

Mean air temperature at the Juneau airport across the six-month study period (May-October, 2013) was 11.8°C, well above the long term average of 10.8°C, with June and August showing the largest departures above temperature normals. Rainfall for the study period was 904 mm, which was comparable to the long term average of 870 mm at the Juneau airport. Stream physical characteristics (temperature, turbidity and DO) were measured at 1 hour intervals using a YSI Sonde (model 6600) mounted vertically on a t-post driven into the streambed at both Peterson (58.5°N, -134.8°W) and Cowee Creek (58.7°N, -134.9°W). Manual measurements using a hand-held YSI (model 556) and HACH (2100P) turbidimeter were made twice a week to check the high resolution measurements obtained from the YSI Sondes.

Discharge in Cowee Creek was measured at 15-minute intervals across the study period using a stilling well equipped with a pressure transducer (In Situ, Troll 500). The stage-discharge relationship was used to calculate streamflow. Discharge in Peterson Creek is measured by the Alaska Department of Fish and Game. Grab samples for water isotope (δ^18^O) analysis were collected at each site twice a week in glass bottles with zero headspace. Streamwater δ^18^O values can be used to assess seasonal differences in the source of streamflow because snow and glacier ice are typically depleted in δ^18^O relative to groundwater and rainfall [31,32]. Water isotope analysis was performed on a Picarro L2120-I analyzer within one month of collection. The δ^18^O values are reported in per mil (‰) following normalization to Vienna standard mean ocean water (VSMOW).

We used visual observations and total dissolved nitrogen (TDN) concentrations as an indicator of spawning salmon abundance based on previous research showing that [TDN] is strongly correlated with the presence of salmon carcasses in Peterson Creek [29]. Streamwater grab samples for TDN were collected at least five days per week across the study period from automated water samplers (ISCO) installed on the stream bank near both monitoring stations. Water samples for TDN were immediately filtered upon collection (every 2–4 days) through pre-combusted Whatman GF/F filters (nominal pore size of 0.7 μm) and concentrations were measured via high temperature combustion on a Shimadzu TOC-V CSH-TN analyzer. A mean concentration and standard deviation were generated for the entire study period and values greater than one standard deviation above the mean were considered days where salmon carcasses were abundant in the stream. Using this approach, we estimated that from August 7–31 salmon carcasses were abundant most days in both streams. We used the maximum weekly average temperature (MWAT) as an indicator of stream thermal suitability for salmon [8,33,34]. The MWAT is commonly used temperature criterion in risk assessments of salmon streams because it is a simple and cost effective technique for temperature tolerance estimates (e.g.,[35]).

### Regression models to assess controls on stream temperature and dissolved oxygen

We used multiple linear regression (MLR) to assess the influence of air temperature and stream discharge on mean monthly stream temperature (mean of daily temperature from hourly sample intervals averaged over 1 month). We also used MLR to assess how stream temperature and discharge influenced mean monthly DO concentration (mean of daily DO levels from hourly sample intervals averaged over 1 month). Stepwise regression analysis using SPSS software was used for each monthly MLR model, and only those variables significant at P<0.05 were retained. Furthermore, linear regression was used to assess the relationship between mean daily stream temperature and DO using SPSS software. Previous research at both study sites has shown that nonlinear regression models of stream temperature versus air temperature did not improve the fit due to the narrow temperature range of our study streams [8].

## Results

### Watershed hydrology

**Fig 2:**
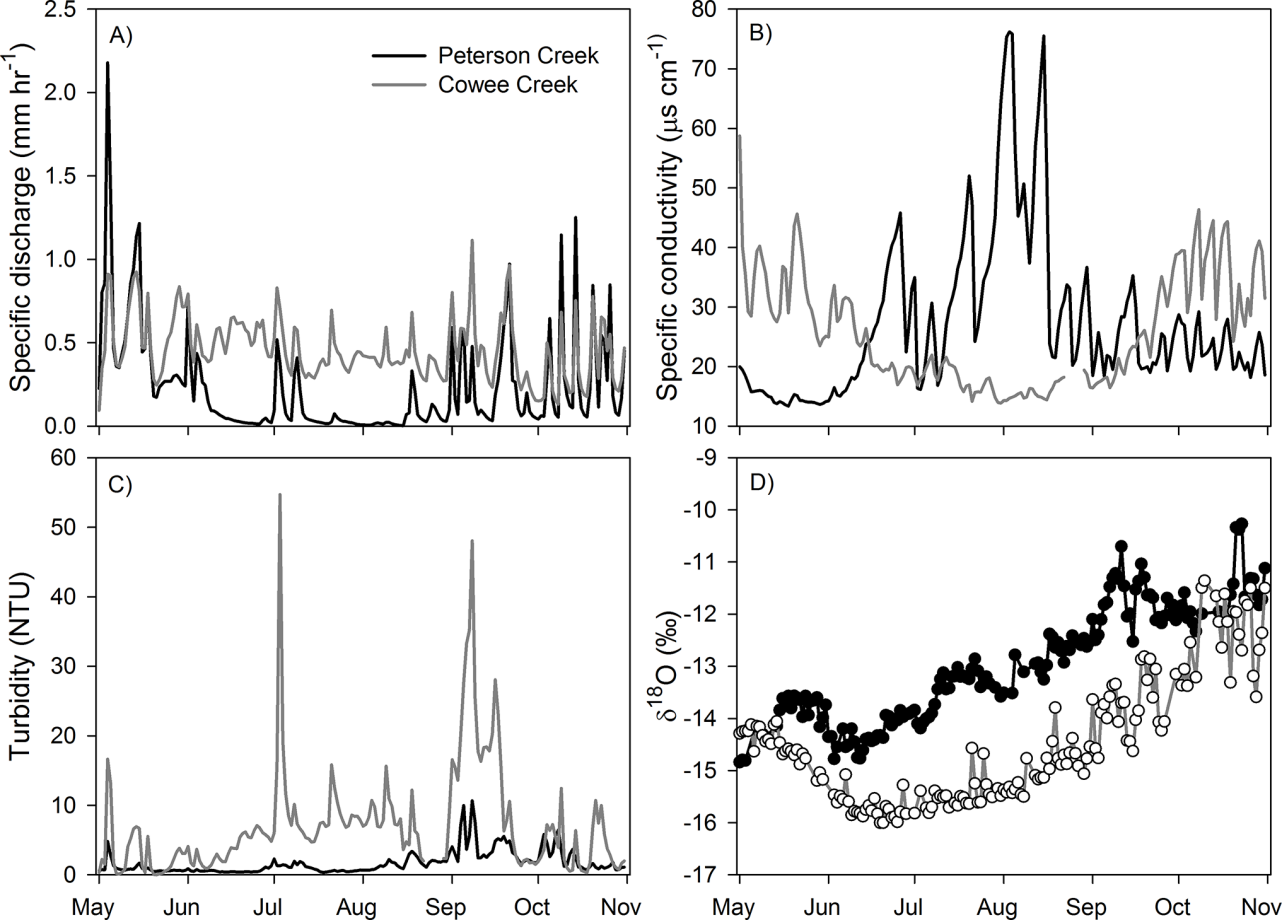
Time series of A) mean daily specific discharge (mm hr^-1^), B) δ^18^O values (‰), C) specific conductivity (μs cm^-1^) and D) turbidity (NTU) for the two study watersheds during the 1 May through 31 October, 2013 study period.

Non-glacial Peterson Creek had a mean daily specific discharge (i.e. runoff) that ranged from <0.01 to 2.18 mm hr^-1^ showing many rainfall spikes across the sample period (Fig 2A; Table 1; S1 Table). Runoff in Peterson Creek also varied seasonally, with an overall decrease from early May to its summer minimum in late July and an increase during the autumn rainy season. Mean daily runoff in glacial-fed Cowee Creek ranged from 0.09 to 1.12 mm hr^-1^ showing less variability relative to Peterson Creek (Fig 2A). The coefficient of variation for mean daily runoff was 0.41 for Cowee Creek compared to 1.35 for Peterson Creek.

**Table 1:** Summary statistics for mean monthly (±1 standard deviation) physical parameters for Peterson and Cowee Creeks for the 1 May through 31 October, 2013 study period.

	Month	Spec Cond μs cm^-1^	Turbidity NTU	Stream temp °C	DO mg L^-1^	Discharge m^3^ s^-1^	δ^18^O ‰
Peterson	May	15.3 (2.3)	1.0 (0.9)	3.7 (2.4)	13.3 (1.0)	3.8 (3.0)	-14.0 (0.4)
Creek	June	25.0 (9.7)	0.5 (0.2)	12.0 (3.3)	9.7 (1.6)	0.8 (1.1)	-14.3 (0.3)
	July	32.1 (10.7)	1.0 (0.5)	13.6 (0.7)	8.4 (1.0)	0.6 (0.9)	-13.5 (0.4)
	Aug	44.8 (18.9)	1.6 (0.7)	13.8 (0.8)	6.6 (1.3)	0.4 (0.5)	-12.8 (0.4)
	Sep	23.7 (4.5)	4.0 (2.3)	11.2 (1.8)	9.4 (1.1)	1.7 (1.6)	-11.8 (0.5)
	Oct	23.2 (3.1)	2.2 (1.6)	7.3 (0.7)	11.4 (0.6)	2.1 (2.2)	-11.7 (0.6)
Cowee	May	34.0 (7.6)	3.0 (3.9)	3.2 (1.1)	13.0 (0.3)	17.2 (7.0)	-14.5 (0.3)
Creek	June	23.7 (5.0)	3.8 (2.0)	6.4 (1.2)	12.1 (0.4)	16.3 (3.1)	-15.7 (0.2)
	July	17.8 (2.3)	10.0 (9.0)	7.5 (0.6)	11.7 (0.3)	13.2 (4.1)	-15.5 (0.3)
	Aug	16.0 (1.5)	7.0 (3.4)	8.1 (0.5)	11.4 (0.2)	12.1 (2.7)	-14.9 (0.4)
	Sep	24.1 (6.6)	15.2 (11.8)	7.3 (0.7)	11.7 (0.1)	14.8 (7.3)	-13.8 (0.6)
	Oct	36.3 (6.2)	3.9 (3.4)	5.8 (0.6)	12.1 (0.2)	10.8 (5.9)	-12.4 (0.7)

Streamwater δ^18^O values averaged -13.0‰ for Peterson Creek and -14.5‰ for Cowee Creek across the study period reflecting differences in the relative contributions of glacial meltwater and precipitation to streamflow (Fig 2B; Table 1). Values for δ^18^O in Peterson Creek were the most depleted (-14.5 to -15.0‰) in early May and again in early June but increased from mid-June through early September. In contrast, Cowee Creek had δ^18^O values below -15.0‰ through most of August reflecting the contribution of glacier meltwater (average = -16.4‰; [8]) to streamflow, with values only becoming more similar to the seasonal mean δ^18^O signature of rainfall (average = -11.4±0.7‰) in October.

### Streamwater physical characteristics

Mean specific conductivity for the study period was comparable between the two watersheds (27.4 μS cm^-1^ in Peterson Creek and 25.6 μS cm^-1^ in Cowee Creek) despite showing dramatically different seasonal patterns (Fig 2C; Table 1; S1 Table). Conductivity in Peterson Creek ranged from 15 to 20 μS cm^-1^ in late spring/early summer, increased substantially to its mid-summer maximum (76.2 μS cm^-1^) in early August, and then declined sharply in mid-August, with values generally <30 μS cm^-1^ for the remainder of the monitoring period. In contrast, specific conductivity in Cowee Creek was highest during the spring (May) and fall (Sep-Oct) and consistently low (<25 μS cm^-1^) during the main glacial runoff period of July and August. Streamwater turbidity was substantially lower in non-glacial Peterson Creek relative to Cowee Creek across the study period (Fig 2D; Table 1; S1 Table). The highest turbidities in Peterson Creek occurred during the fall rainy months of September and October. Cowee Creek had highly variable turbidities reflecting inputs of glacial silt during the summer runoff period and runoff from the forested landscape during large rainstorms.

**Fig 3:**
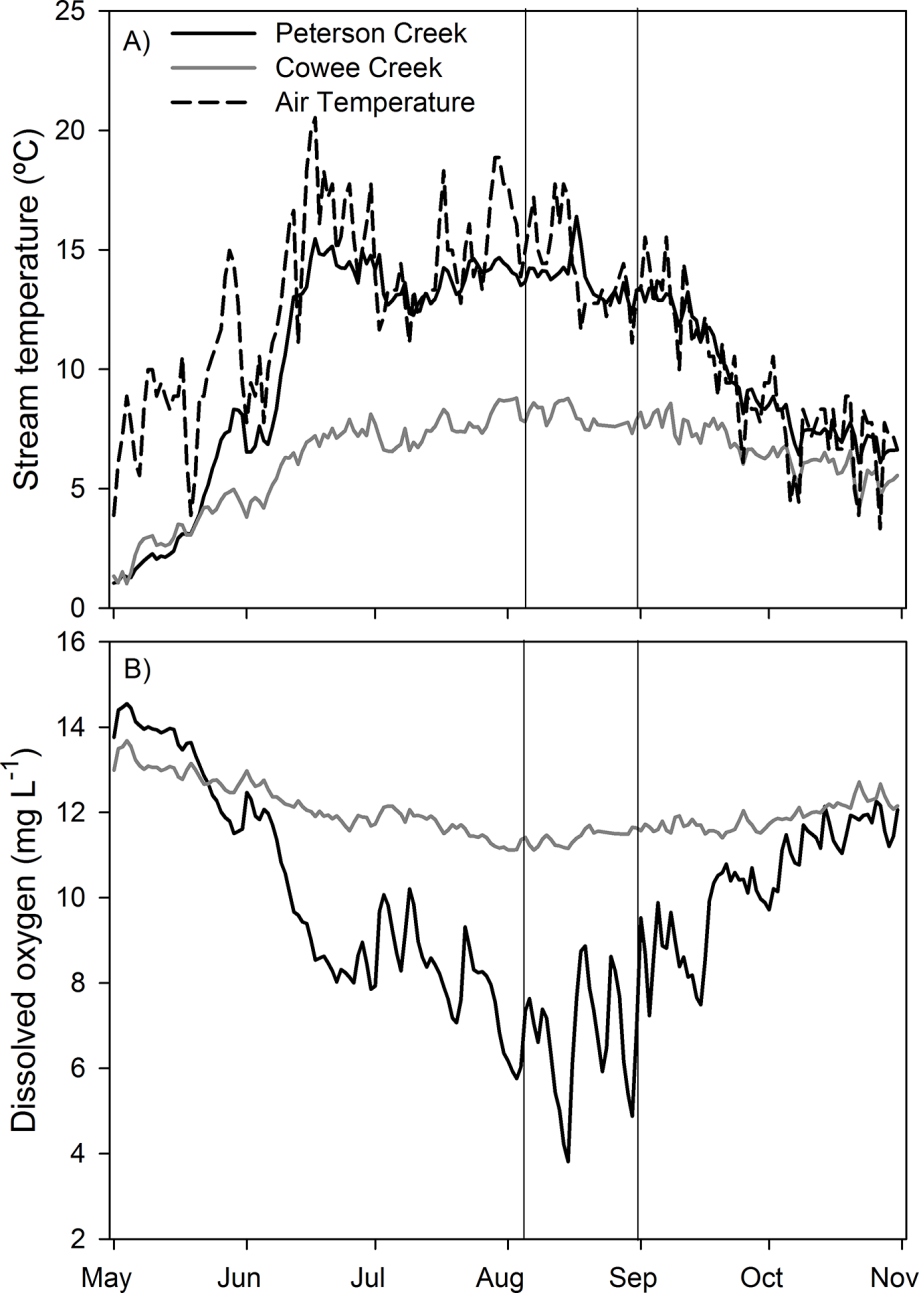
Time series of A) mean daily stream and air temperature and B) DO for the two study watersheds during the 1 May through 31 October, 2013 study period. The two vertical solid lines correspond to the period of time when spawning salmon were abundant in the study streams.

Mean daily stream temperature in Peterson Creek ranged from 1.1 to 16.4°C across the study period and demonstrated high variability relative to Cowee Creek, where temperature ranged from only 1.0 to 8.8°C (Fig 3A; Table 1; S1 Table). Stream temperature in both watersheds generally tracked air temperatures, although the pattern was strongly muted in Cowee Creek compared to Peterson Creek. The MWAT, which was used to evaluate thermal habitat quality for salmon, was 14.9°C for Peterson Creek and 8.6°C for Cowee Creek. The MWAT occurred in early August in Cowee Creek coinciding with the period of time spawning salmon were in the stream (Fig 3A). In Peterson Creek, the MWAT occurred in mid-June (16–22 of June) coincident with the warmest air temperatures of the summer.

Mean daily concentrations of DO in Peterson Creek ranged from 3.8 to 14.1 mg L^-1^ showing dramatic seasonal variation across the study period (Fig 3B; Table 1; S1 Table). Mean daily DO in Peterson Creek was lowest (3.8 to 8.9 mg L^-1^) during August when discharge was generally low and spawning salmon were abundant in the stream and highest during May and October when stream temperature was lowest (Fig 3B). In contrast, mean daily concentrations of DO in Cowee Creek showed relatively little seasonal variation across the study period ranging from 11.1 to 13.2 mg L^-1^.

### Seasonal controls on stream temperature and dissolved oxygen

**Fig 4:**
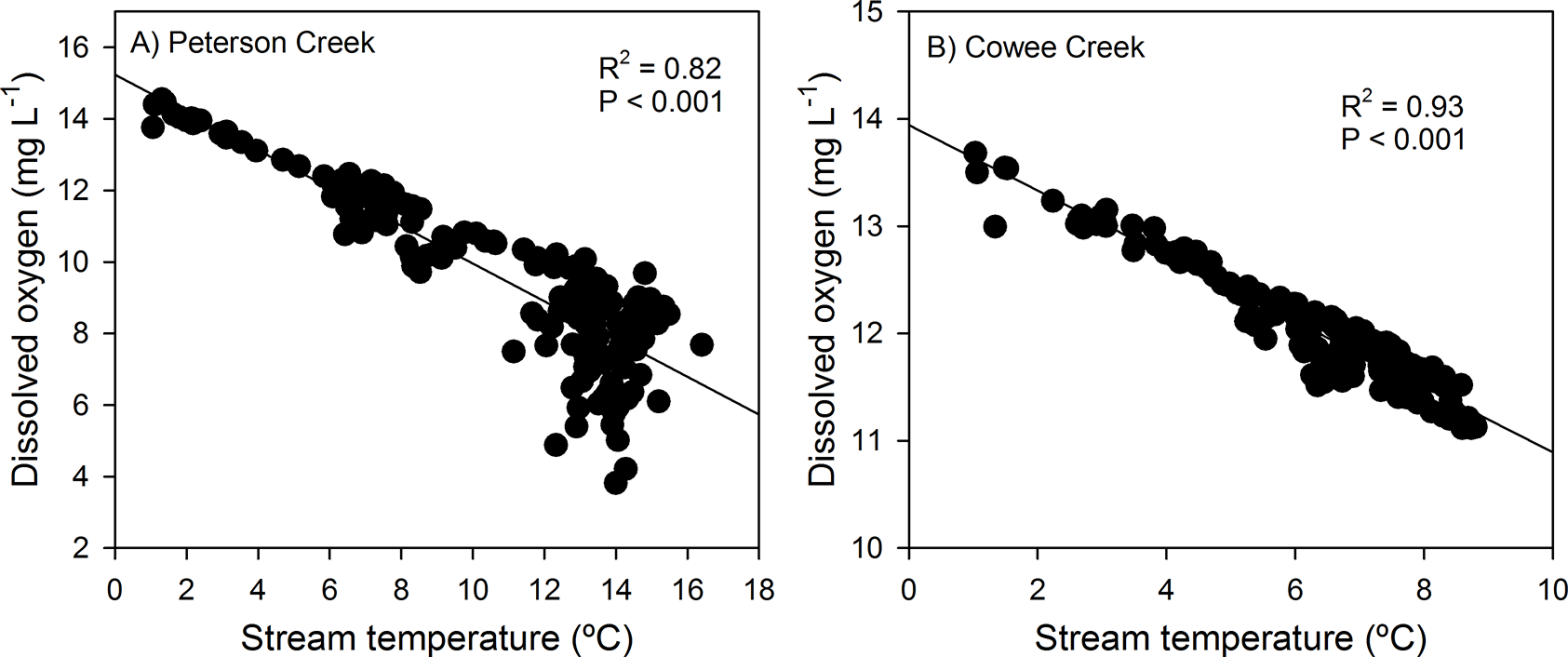
Regression models describing the relationship between mean daily stream temperature and mean daily DO in A) Peterson Creek and B) Cowee Creek during the 1 May through 31 October, 2013 study period.

**Fig 5:**
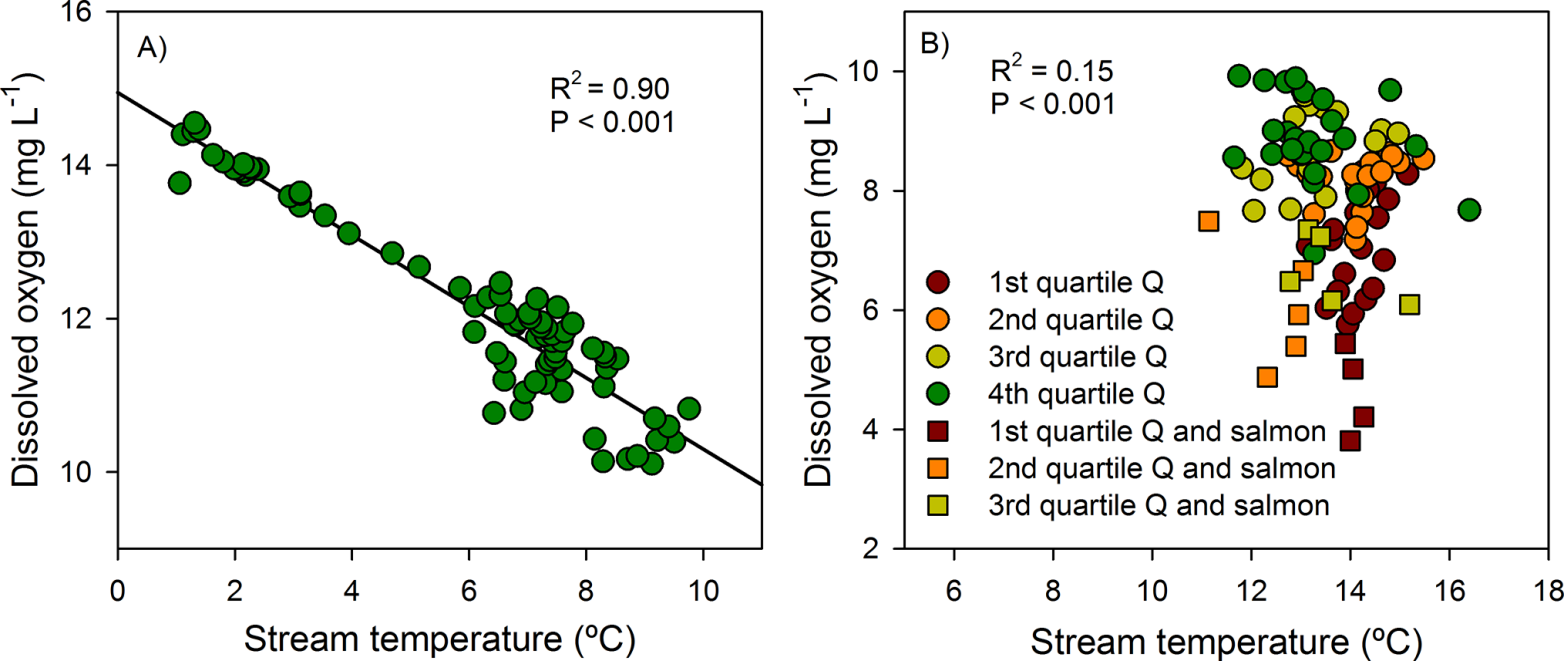
Regression models in Peterson Creek describing the relationship between mean daily stream temperature and mean daily DO A) on days when stream temperature was < 10°C and B) on days when stream temperature was >10°C. The four discharge (Q) quartiles divide mean daily discharge into four quarters and “salmon” refers to days when spawning salmon carcasses were abundant in the stream.

Mean daily stream temperature was strongly correlated with mean daily DO in both Peterson (R^2^ = 0.82, P<0.001; Fig 4A) and Cowee Creek (R^2^ = 0.93, P<0.001; Fig 4B). However, this correlative relationship in Peterson Creek was driven by the highly significant (R^2^ = 0.90, P<0.001) relationship between DO concentration for stream temperatures <10°C (Fig 5A). On days when stream temperatures were >10°C, DO was mildly related to stream temperature (R^2^ = 0.15, P>0.001) and relatively strongly influenced by discharge (R^2^ = 0.46, P<0.001; Fig 5B). These results suggest that the relationship between DO and stream temperature/discharge is best expressed by a piecewise regression, with a stream temperature break point of ~10°C, below which DO concentrations are influenced by stream temperature and above which discharge is a stronger control. Interestingly, concentrations of DO in Peterson Creek were particularly low (<5.0 mg L^-1^) on days discharge was low and spawning salmon were abundant (Fig 5B).

Multiple linear regression models showed that air temperature and discharge were strong predictors of mean monthly stream temperature in both Peterson and Cowee Creeks (Table 2). However, in some months (e.g., July and September), discharge did not improve the model fit leaving air temperature alone as the significant predictor of stream temperature at both sites. The most robust stream temperature models in Peterson Creek were typically the spring and fall months (except October), while in Cowee Creek the model was strongest in June-Sept. Mean monthly DO concentrations in Peterson Creek were significantly related to stream temperature and/or discharge for all months, with the strongest (May) and weakest (August) models explaining 99% and 45% of the variance, respectively (Table 2). In Cowee Creek, stream temperature and to a lesser extent discharge were strong predictors of mean monthly DO concentrations in all months except September.

**Table 2:** Summary statistics for multiple linear regression models used to predict mean monthly stream temperature (air temperature and discharge are predictor variables) and mean monthly DO (discharge and stream temperature are predictor variables) for the 1 May through 31 October, 2013 study period.

	Month	Stream temp R^2^	Stream temp SE	DO R^2^	DO SE
Peterson	May	0.71*	1.31	0.99*	0.10
Creek	June	0.84*	1.37	0.95*	0.35
	July	0.40* ^,^ ^A^	0.60	0.68*	0.56
	Aug	0.37†	0.68	0.45* ^,^ ^Q^	0.98
	Sep	0.80* ^,^ ^A^	0.84	0.76*	0.53
	Oct	0.43* ^,^ ^A^	0.54	0.70*	0.35
Cowee	May	0.58*	0.77	0.88* ^,^ ^S^	0.11
Creek	June	0.81* ^,^ ^A^	0.55	0.97* ^,^ ^S^	0.06
	July	0.73* ^,^ ^A^	0.33	0.79* ^,^ ^S^	0.14
	Aug	0.78*	0.23	0.88* ^,^ ^S^	0.07
	Sep	0.85* ^,^ ^A^	0.27	0.25	0.13
	Oct	0.44*	0.49	0.78*	0.12

* indicates P<0.001

† indicates P<0.01

A indicates the model is significant with air temperature only

S indicates the model is significant with stream temperature only

Q indicates the model is significant with discharge only.

## Discussion

### Watershed hydrology

Non-glacial Peterson Creek had high variability in specific discharge across the study period relative to glacial-fed Cowee Creek, emphasizing how runoff from mountain glaciers attenuates seasonal variability in discharge [23,24,36]. The patterns in discharge were consistent with the δ^18^O signatures for both streams, where snow and glacial meltwater inputs to Cowee Creek resulted in δ^18^O values remaining depleted (<-15.0‰) well into August. In contrast, δ^18^O values in Peterson Creek increased during this same period and became more similar to the δ^18^O signature of rainfall in the study area [8]. The winter snowpack in Peterson watershed was mostly ablated by early summer, leaving rainfall/groundwater as the main sources of streamflow for the remainder of the monitoring period. Together, these results elucidate how seasonally evolving sources of streamwater influence the timing and magnitude of discharge and highlight how watershed hydrology differs between non-glacial streams, where discharge is driven by summer precipitation, and glacial streams, where discharge is strongly influenced by the energy balance at the glacier surface [23,37].

### Controls on seasonal stream temperature and dissolved oxygen

The results of our multiple regression analysis for mean monthly stream temperature are consistent with other studies of forested watersheds showing that air temperature [38,39] and to a lesser extent discharge [7,40] are moderate to strong controls on seasonal stream temperature. However, for some months (e.g., August and October) when discharge and air temperature are only mildly related to stream temperature, other variables such as watershed landcover are likely important. For instance, glacier coverage and lake area have been shown to be tightly linked to stream temperature in Southeastern [8] and Southcentral Alaska [6,41] as well as other regions [21,26,42]. Glaciers are a particularly strong landscape control on stream temperature, with previous studies of proglacial streams observing a decrease in water temperature of 1.1 to 1.2°C for each 10% increase in glacier coverage [8,21]. Thus, glacier meltwater provides a thermal buffering relative to non-glacial streams [8,21,43] that might become increasingly important in the coming decades given projected regional changes in air temperature (increase of >2C° by 2080s) and the areal coverage of seasonal snow (decrease of 22–58% by 2080; [44]).

Seasonal concentrations of DO in Cowee Creek showed little variability across the study period suggesting that glacier meltwater had a stabilizing effect on DO by moderating stream temperature (always <10°C) and maintaining streamflow volume, thus preventing the seasonal depletion of DO observed in non-glacial Peterson Creek. Concentrations of DO in Peterson Creek were strongly related to stream temperature below 10°C (R^2^ = 0.90, P<0.001), but were mildly related to stream temperature (R^2^ = 0.15, P<0.001) and strongly related to discharge (R^2^ = 0.46, P<0.001) when temperature exceeded 10 ºC. During periods of low flow, reduced streamwater turbulence can contribute to DO depletion by decreasing the vertical mixing that facilitates streamwater oxygenation [19]. In addition, low flow periods increase the thermal sensitivity of the stream to elevated air temperatures by decreasing the thermal mass of streamwater. The impacts of reduced mixing and elevated stream temperatures associated with reduced streamflow are additive in terms of reducing streamwater DO. In Peterson Creek, the % saturation of streamwater DO was regularly below 75% on days when stream temperature exceeded 10°C and streamflow was relatively low (<20 cfs), but close to saturation when temperature was below 10°C. These results highlight the idea that future DO depletion events in small coastal streams will likely be driven by changes in hydrology in addition to atmospheric warming.

The seasonal DO depletion observed in Peterson Creek also appears to be influenced by biotic factors. Maximum DO depletion occurred during mid-August when discharge was extremely low and spawning salmon (live and dead) were abundant based on streamwater TDN concentrations. Ecosystem respiration (ER) in salmon streams of Alaska has been shown to peak when spawning salmon densities are highest likely a result of salmon themselves stimulating ER [45,46]. Moreover, this salmon-induced spike in ER can result in a dramatic decrease in DO [45] suggesting elevated ER driven by the presence of salmon likely contributed to DO depletion in Peterson Creek during August particularly on days when streamflow was low. Spawning salmon did not appear to have any impact on DO concentrations in Cowee Creek, likely because of the substantially lower density of spawning fish relative to streamflow volume. Overall, our results suggest that multiple interacting physical (stream temperature and discharge) and biotic (salmon) factors control seasonal DO patterns in Peterson Creek.

### Stream habitat quality for Pacific salmon

Stream temperature and DO regimes are strong controls on potential freshwater habitat quality for salmon. Controlled laboratory studies and growth models for salmon at various life stages have produced well-defined physiological tolerances for temperature and DO [47–49]. However, these standards may not be directly applicable to evaluate salmon habitat quality because stream ecosystems exhibit some of the most dynamic habitat conditions in nature [50] and salmon are able to adjust to spatio-temporal changes in habitat variability by migrating to more favorable habitat when necessary [13,51,52]. For instance, juvenile salmon have been shown to occupy different thermal habitats depending on streamflow [52] and the presence of zones of DO depletion [13] or to increase their assimilative capacity [14]. Moreover, our data are limited in temporal and spatial extent and thus, our results represent a first approximation of suitable freshwater habitat for salmon.

There were clear inter-stream differences in potential habitat quality for salmon. In particular, the optimal range in MWAT for salmon development is12.8–14.8°C based on growth metrics for salmon at different life stages [48]. The MWAT we measured for Cowee Creek (8.6°C) falls 4.2°C below the optimal range for salmon indicating that on a regional basis, glacier runoff can contribute to sub-optimal physical habitat (e.g., cold and turbid) for salmon [8,53,54]. In contrast, Peterson Creek was at the high end of the optimal range for salmon physiology consistent with other studies in Southeastern [8] and Southcentral Alaska [6,41] showing that low gradient forested watersheds currently provide favorable thermal habit for salmon. However, the regression slope of mean daily stream temperature against mean daily air temperature in Peterson Creek was 0.78, indicating fairly high sensitivity to future increases in air temperature [38].

In Peterson Creek, there were four days where mean daily DO was <5 mg L^-1^, which is the threshold where DO levels generally start to impact salmon behavior (e.g., avoidance; [51,55,56] and metabolic activity [57,58] as well as prevent upstream migration [17]. Concentrations of DO below 5.0 mg L^-1^ have also been reported to cause mortality and negatively impact aquatic macroinvertebrate communities [19,59]. Therefore, low DO concentrations could impact freshwater habitat quality for salmon directly and also indirectly through increased macroinvertebrate mortality, which is likely an important food source for juvenile coho (*O*. *kisutch*) salmon in the stream. Although the low DO episodes observed during August in Peterson Creek were likely too short in duration to cause dramatic impacts to aquatic habitat quality, these values are bordering the critically low range such that climate warming that results in an increase in the frequency and duration of DO depletion could cause sub-lethal and potentially lethal impacts to aquatic species. For instance, if climate change causes summertime stream temperatures to increase only slightly or if more extensive periods of low flow become more common in lower elevation forested streams like Peterson Creek (e.g.,[60]), DO levels could fall into the critically low range for more extensive periods of time, degrading habitat quality for aquatic species. Moreover, salmon-induced spikes in stream ER could result in depressed DO values when spawner densities are high (e.g., [45]), although this is most likely an additive impact during periods of low streamflow rather than a top-down control on stream DO. Ultimately, developing quantitative models that allow for projections of stream temperature and hydrologic conditions and their interactions with each other and biotic factors (e.g., elevated ER when spawning salmon are present), are clearly necessary for understanding how freshwater habitats that support salmon in the region will be impacted by climate warming.

### Future changes in aquatic ecosystems

Coastal Mountain glaciers of southeast Alaska are currently experiencing high rates of mass loss [61,62] and are projected to continue losing mass at rapid rates in the future [63]. Future decreases in glacier volume will impact streamflow regimes by driving a transient increase in meltwater inputs to streamflow (cooling stream temperature) that is followed by a long-term decrease in runoff [23,24,36]. Our findings suggest that as glacier runoff declines and streams undergo a shift in the dominant source of streamwater towards seasonal snowmelt and rainfall, more variable temperature and DO regimes will prevail [64,65]. The greatest changes in discharge and stream physical properties are likely to occur in mid to late summer following melting of seasonal snowpacks, as exemplified by the extended periods of low flow and DO depletion in non-glacial Peterson Creek during mid-July through August. This period of time corresponds to the peak of salmon spawning in watersheds along the Gulf of Alaska. Taken together, our findings support the notion that some salmon runs (heavily glaciated watersheds) might benefit from future climate warming but others (low gradient forested watersheds) might experience declines [66,67].

Climate-driven changes in streamflow timing [9,60] have the potential to dramatically alter the biophysical characteristics of stream ecosystems. While there has been considerable attention aimed at understanding the effects of flow reduction on upstream salmon migration [68] and macroinvertebrate communities [69], less attention has focused on the effects of flow reduction on DO regimes (except see [19]). Our findings suggest that severe low flows may also indirectly affect aquatic species through DO depletion caused by decreased vertical mixing of oxygen. Moreover, decreasing streamflow would be accompanied by a decrease in streamwater albedo thereby facilitating greater in-stream absorption of solar radiation and increasing stream temperature [70].

Stream temperature response to climate warming has become a pressing environmental issue in some regions of the world, such as the European Alps and the Pacific Northwest of the USA because warmer temperatures are already negatively impacting available habitat for cold-water fish species [67,71,72]. Although the health of salmon runs are not determined from individual factors alone (e.g., tradeoff between food availability, physiology and physicochemical properties; [73]), standards are necessary for scientists and managers to evaluate the potential effects of climate warming on salmon streams. Our finding that DO regimes can result from interacting physical and biologic controls highlights the complexity of salmon habitat abiotic factors in coastal temperate watersheds. Overall, our results highlight the need for watershed managers to move towards multi-factor risk assessment of potential freshwater habitat quality for salmon rather than single factor assessments alone.

## Supporting Information

S1 Table Mean daily values for air temperature, dissolved oxygen, stream temperature, discharge, turbidity and specific conductivity in both Cowee and Peterson Creeks. (TXT)
